# Automated Quantification of Extranuclear ERα Using Phosphor-Integrated Dots for Predicting Endocrine Therapy Resistance in HR^+^/HER2^−^ Breast Cancer

**DOI:** 10.3390/cancers11040526

**Published:** 2019-04-12

**Authors:** Zhaorong Guo, Hiroshi Tada, Narufumi Kitamura, Yoh Hamada, Minoru Miyashita, Narumi Harada-Shoji, Akiko Sato, Yohei Hamanaka, Kouki Tsuboi, Nobuhisa Harada, Mayumi Takano-Kasuya, Hisatake Okada, Yasushi Nakano, Noriaki Ohuchi, Shin-ichi Hayashi, Takanori Ishida, Kohsuke Gonda

**Affiliations:** 1 Department of Breast and Endocrine Surgical Oncology, Graduate School of Medicine, Tohoku University, Sendai, Miyagi 980-8574, Japan; guozhaorong1@yahoo.co.jp (Z.G.); atihsayim8m8@med.tohoku.ac.jp (M.M.); vecchio03@yahoo.co.jp (N.H.-S.); aki_aki_tan@yahoo.co.jp (A.S.); h-y@med.tohoku.ac.jp (Y.H.); noriaki-ohuchi@med.tohoku.ac.jp (N.O.); takanori@med.tohoku.ac.jp (T.I.); 2 Department of Medical Physics, Graduate School of Medicine, Tohoku University, Sendai, Miyagi 980-8574, Japan; n-kitamura@med.tohoku.ac.jp (N.K.); yhamada@med.tohoku.ac.jp (Y.H.); takano@med.tohoku.ac.jp (M.T.-K.); gonda@med.tohoku.ac.jp (K.G.); 3 Department of Molecular and Functional Dynamics, Graduate School of Medicine, Tohoku University, Sendai, Miyagi 980-8574, Japan; k.tsuboi@med.tohoku.ac.jp (K.T.); shin@med.tohoku.ac.jp (S.-i.H.); 4 Bio Systems Development Group, Bio Advanced Technology Division, Corporate R&D Headquarters, KONICA MINOLTA, INC., Hino, Tokyo 191-8511, Japan; nobuhisa.harada@konicaminolta.com (N.H.); hisatake.okada@konicaminolta.com (H.O.); yasushi.nakano@konicaminolta.com (Y.N.)

**Keywords:** breast cancer, estrogen receptor α, IHC-PIDs, prognostic, endocrine therapy resistance

## Abstract

In addition to genomic signaling, Estrogen receptor alpha (ERα) is associated with cell proliferation and survival through extranuclear signaling contributing to endocrine therapy (ET) resistance. However, the relationship between extranuclear ERα and ET resistance has not been extensively studied. We sought to measure extranuclear ERα expression by immunohistochemistry using phosphor-integrated dots (IHC-PIDs) and to assess its predictive value for ET resistance. After quantitative detection of ERα by IHC-PIDs in vitro, we developed “the nearest-neighbor method” to calculate the extranuclear ERα. Furthermore, tissue sections from 65 patients with HR+/HER2- BC were examined by IHC-PIDs, and the total ERα, nuclear ERα, extranuclear ERα PIDs score, and ratio of extranuclear-to-nuclear ERα (ENR) were measured using the novel method. We demonstrate that quantification of ERα using IHC-PIDs exhibited strong correlations to real-time qRT-PCR (*r*^2^ = 0.94) and flow cytometry (*r*^2^ = 0.98). High ERα ENR was significantly associated with poor overall survival (*p* = 0.048) and disease-free survival (DFS) (*p* = 0.007). Multivariate analysis revealed that the ERα ENR was an independent prognostic factor for DFS [hazard ratio, 3.8; 95% CI, 1.4–11.8; *p* = 0.006]. Our automated measurement has high accuracy to localize and assess extranuclear ERα. A high ERα ENR in HR^+^/HER2^−^ BC indicates decreased likelihood of benefiting from ET.

## 1. Introduction

Estrogen receptor alpha (ERα) is a member of the ligand-dependent nuclear receptor transcription factor family and plays a critical role in the initiation and development of human breast cancer (BC) [1]. Approximately 70% of BCs express ERα, which is the most important determinant of susceptibility to endocrine therapy (ET) [2,3]. Most ERα-positive BCs are treated effectively with ET, which binds competitively to ERα (tamoxifen and fulvestrant) or deprives the tumor of estrogens (i.e., aromatase inhibitor, AI). However, as many as 40% of hormone receptor (HR)-positive patients and essentially all patients with metastatic disease do not benefit from ET (due to de novo or acquired resistance) [4,5]. Several mechanisms have been proposed to explain this resistance, including ER gene (ESR1) mutations, epigenetic aberrations, and signaling crosstalk [4]. Evidence has accumulated over the last two decades indicating that, in addition to the classical view of ERα as a nuclear HR, an extranuclear (nongenomic, non-nuclear, rapid, or membrane-initiated) signaling pathway is mediated by ERα localized at the plasma membrane and in the cytoplasm [6,7]. Additionally, extranuclear ERα function has been documented to be biologically important in BC cell proliferation. Extranuclear ERα mediates endocrine resistance via direct or indirect activation by ERα in response to estrogen release via growth factors (GFs) and receptor tyrosine kinases (RTKs) and through additional signaling and coactivator molecules. This interaction, similar to GF activation of these pathways, activates multiple downstream kinase pathways (e.g., SRC, PI3K/AKT/mTOR and Ras/Raf/ERK) which in turn phosphorylate various transcription factors and coregulators, including components of the ER pathway, that enhance gene expression on estrogen response elements (EREs) and other response elements [8,9,10]. Additionally, a previous study indicated that low concentrations of estradiol can stimulate proliferation of long-term estradiol deprivation of cells, in contrast to MCF-7 cells. Thus, AI resistance can evolve due to ER activation via ER hypersensitivity and ligand-independent ER activation by activated GF signaling [11]. 

Clinically, only nuclear ERα is measured using immunohistochemistry (IHC) staining with the chromogen dye diaminobenzidine (DAB), whereas extranuclear ERα is missed due to its sparse localization [12,13]. The few studies conducted on this subject were almost exclusively performed in cell line models [14], and only one study showed that ERα can be observed in the cytoplasm of BC specimens using AQUA technology (HistoRx Inc. CT, USA) [15]. Unfortunately, due to the low detection rate of cytoplasmic ERα, the study failed to demonstrate whether extranuclear ERα could predict endocrine resistance. Thus, due to the low quantitative sensitivity of IHC-DAB and AQUA, the detection of cytoplasmic ERα is less likely be widely applied in routine clinical practice.

Recently, many researchers have shown interest in the application of fluorescent nanoparticles known as quantum dots (Qdots) in the quantitative diagnosis of BC due to their high photostability and brightness [16,17,18,19]. However, quantitative IHC-based analysis remains challenging due to strong tissue autofluorescence, resulting in an intensity comparable to Qdot fluorescence intensity. To mitigate these problems, our laboratory recently developed nanoparticles suitable for IHC called phosphor-integrated dots (PIDs). PIDs are packed with an organic fluorophore (pelylene diimide) at high density, and the fluorescence intensity after excitation at 580 nm is approximately 100-fold greater than that of Qdot 625. Therefore, PIDs have significantly stronger fluorescence signals than tissue autofluorescence and can be recognized at the single-particle level. PIDs also have a wider dynamic range, from very low to fairly high, than that of conventional fluorescent dyes, Qdots, and DAB [20,21,22]. PIDs retain morphological information, and thus extranuclear and nuclear ERα localization can be visualized and quantitatively analyzed by this method.

SP1 is a rabbit monoclonal antibody (mAb) against the COOH-terminal region of ERα, and 1D5 is a mouse monoclonal antibody against the N-terminal region of ERα. SP1 is widely used in clinical diagnosis of BC and has been reported to be more sensitive than 1D5 [23,24]. Additionally, a previous study convincingly visualized extranuclear ERα in a cell model and indicated that an antibody recognizing the COOH-terminal sequence of ERα could recognize >90% of ERα in the membrane [14]. Thus, SP1, but not 1D5, is suggested to be the appropriate antibody to measure extranuclear ERα.

In the present study, we aimed to explore the significance of extranuclear ERα in predicting the benefit of ET and patient prognosis. We validated the sensitivity and accuracy of IHC with PIDs (IHC-PIDs) for measuring extranuclear ERα. We developed “the nearest-neighbor method” to assess ERα expression levels in the cytoplasm and/or at the membrane in BC specimens, and investigated the relationship between extranuclear ERα and ET resistance in clinical data.

## 2. Results

### 2.1. Quantitative Sensitivity of IHC-PIDs In Vitro

**Figure 1:**
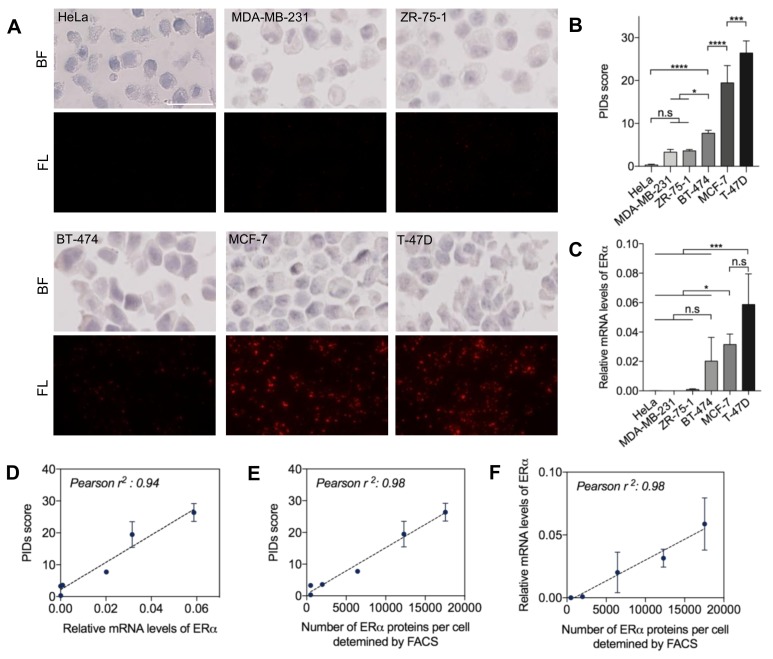
Quantitative sensitivity of immunohistochemistry using phosphor-integrated dots (IHC-PIDs) in vitro. (**A**) Representative imaging of six hormone-associated cancer cell lines stained with haematoxylin and PIDs shows an increasing fluorescent dot-dependent pattern in fluorescence field (FL). Bar, 20 μm. Estrogen receptor alpha (ERα) protein and mRNA expression were compared for each cancer cell line to calculate the PIDs score (PID particles per cell) (**B**) and relative copy number (**C**), respectively (mean±SD, one-way ANOVA with Dunnett’s multiple comparison; * *p* < 0.05, *** *p* < 0.001, **** *p* < 0.0001). (**D**–**F**) Strong correlations between each pair of values (PIDs score and relative mRNA levels of ERα: Pearson *r*^2^ = 0.94; number of ERα protein per cell determined by FACS and PIDs score: Pearson *r*^2^ = 0.98; relative mRNA levels of ERα and number of ERα protein per cell determined by FACS: Pearson *r*^2^ = 0.98).

The lab initially developed IHC-PIDs and an image-processing method that enabled the highly accurate calculation of HER2 expression using the PIDs score [21]. Since ERα, unlike HER2, is mainly localized in the nucleus, we analyzed the quantitative sensitivity of IHC-PIDs for ERα using six cell lines with different ERα protein and mRNA expression levels. Paraffin sections of cultured cells were stained with the primary SP1 antibody, secondary antibody, and PIDs and were observed using a versatile fluorescence microscope (Figure 1A). The PIDs score showed that the expression levels were significantly different among cell lines that highly expressed ERα (MCF-7, and T-47D), moderately expressed ERα (BT-474), a cell line with low expression of ERα (ZR-75-1), and ERα-negative cell lines (MDA-MB-231 and HeLa).

In addition, ERα expression levels were also significantly different among the ERα-positive cell lines, such as between BT-474 and MCF-7 cells and between MCF-7 and T-47D cells (Figure 1B). However, only the differences between T-47D or MCF-7 and BT-474 and those between the ERα-positive and ERα-negative cell lines were significant according to real-time qRT-PCR (quantitative reverse transcription-PCR) analysis (Figure 1C). ERα protein expression in these cell lines was also measured by flow cytometry, which is a good quantitative method for protein analysis but an inferior method for preserving cellular morphological characteristics. The three measurements showed strong correlations with each other, with Pearson *r*^2^ values ranging from 0.94 to 0.98 (Figure 1D–F), demonstrating that IHC-PIDs can detect ERα expression levels with high sensitivity and accuracy.

### 2.2. Identification of the Extranuclear ERα by IHC-PIDs

**Figure 2:**
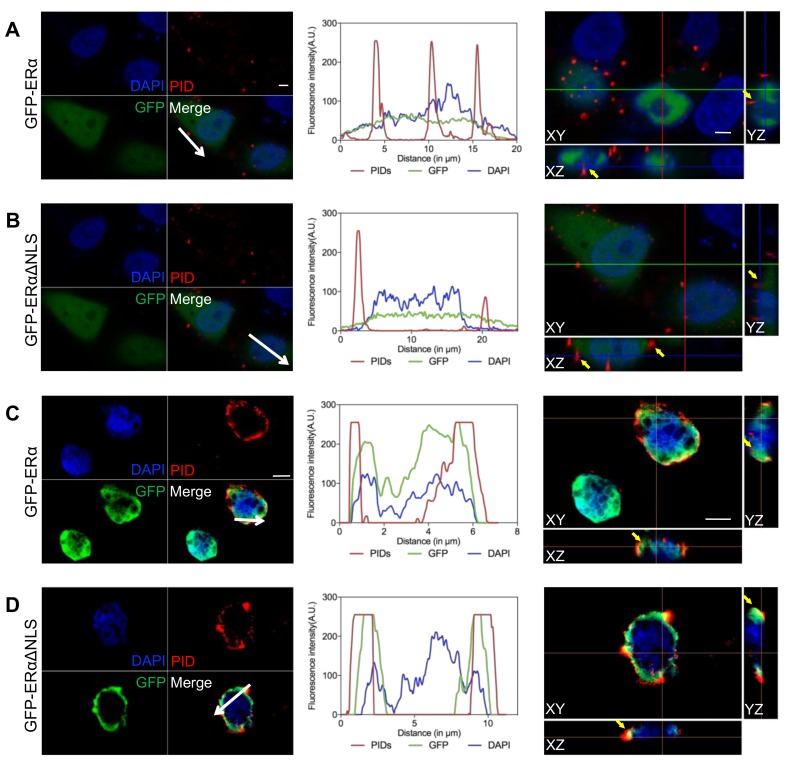
Colocalization studies of PIDs with ERα expression green fluorescent protein (GFP). HeLa cells transfected with GFP-ERα (**A**) or GFP-ERαΔNLS (nuclear localization sequence); (**B**) expression plasmids were observed by confocal microscopy (blue: DAPI; green: GFP; red: PID) (left). Line profile in middle represents the red, green, and blue channel intensities on the sampled white arrow in left. An x–y overview and x–z and y–z slices are shown (right). The yellow arrows illustrating colocalization of PIDs-labelled ERα (red) and ERα expression GFP in nucleus (**A**) or cytoplasm (**B**). Bar, 5 μm. Paraffin sections of GFP-ERα (**C**) or GFP-ERαΔNLS (**D**) expression plasmids-transfected HeLa cells were observed by N-SIM (blue: DAPI; green: GFP; red: PID). The fluorescence intensity profile across the white arrow for red, green, and blue channels is shown in the middle. An x–y overview and x–z and y–z slices are shown (right). The yellow arrows illustrating colocalization of PIDs-labelled ERα (red) and ERα expression GFP in nucleus (**C**) or cytoplasm (**D**). Bar, 5 μm.

To determine whether extranuclear ERα localization could be detected by IHC-PIDs, ERα-negative HeLa cells were transiently transfected with a green fluorescent protein (GFP)-ERαΔNLS (nuclear localization sequence) expression plasmid, and ERα was found to mainly exhibit extranuclear expression. We used control GFP-ERα expression plasmid-transfected HeLa cells that only expressed ERα in the nucleus. After 72 h of transfection, the results of real-time qRT-PCR showed that both plasmids were successfully transfected into HeLa cells (Supplementary Materials, Figure S1). The cells were fixed, permeabilized, stained with IHC-PIDs, and analyzed by optical sectioning and z stack reconstruction using a confocal microscope. As expected, in HeLa cells transfected with the GFP-ERα expression plasmid, PIDs were colocalized with nuclear ERα (Figure 2A). In contrast, in HeLa cells transfected with a GFP-ERαΔNLS expression plasmid, PIDs were mainly colocalized with extranuclear ERα (Figure 2B). 

We stained paraffin embedded sections of transfected HeLa cells with an anti-GFP antibody and PIDs, and the results also showed that PIDs were colocalized with nuclear ERα in GFP-ERα expression plasmid-transfected cells (Figure 2C) and with extranuclear ERα in GFP-ERαΔNLS expression plasmid-transfected cells (Figure 2D). Furthermore, staining for PIDs (red channel) was largely coincident with GFP (green channel) in the fluorescence intensity profile (Figure 2 middle). Notably, even the nuclear membrane was permeabilized with Triton X-100. Because PIDs are as large as 150 nm in diameter, it is relatively difficult for them to enter the nucleus in whole cells (Figure 2A,C). These results suggest that PIDs exhibit appropriate recognition of extranuclear ER in cancer cells.

### 2.3. Validation of the Accuracy of the Nearest-Neighbor Method in Evaluating Extranuclear ERα

**Figure 3:**
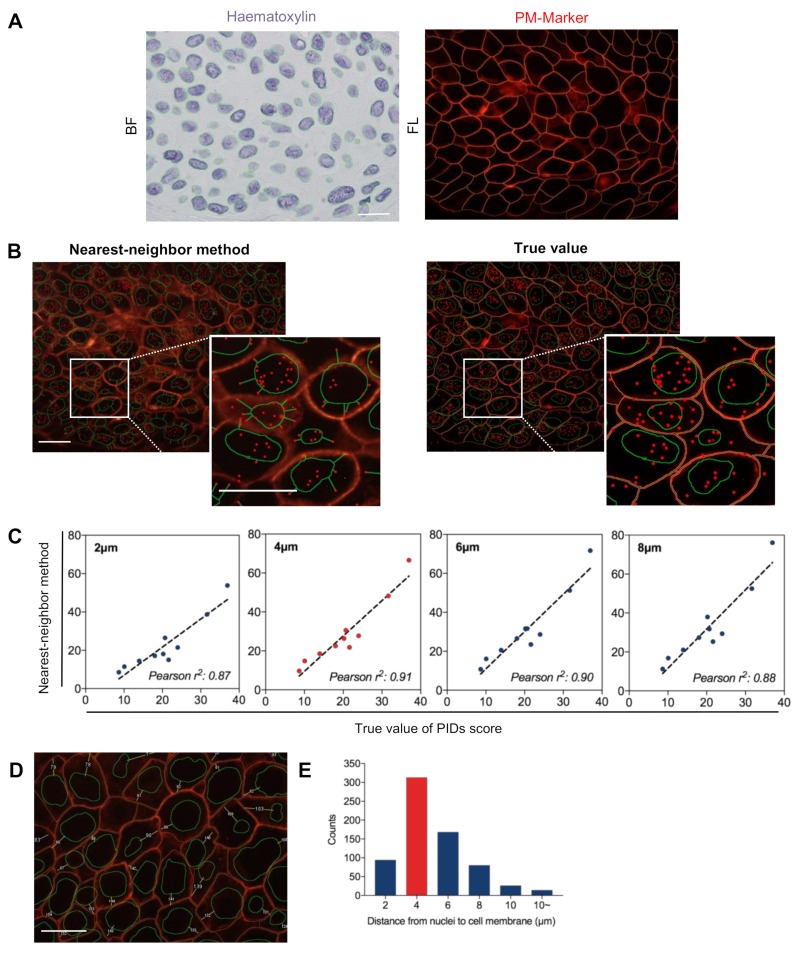
Verification of nearest-neighbor method for the detection of extranuclear ERα. To establish an accurate algorithm, the cell membranes of samples representing a range of ERα expression levels were double stained with haematoxylin (**A**, left) and an Alexa Fluor^®^ 647-labelled plasma membrane marker (PM-Marker) (**A**, right). The nuclear boundary (green line) and cell membrane (red line) both delineated for calculating the true value of extranuclear ERα PIDs score (**B**, right) and then compared to the nearest-neighbor method (**B**, left). (**C**) 4 μm has strongest correlation coefficients between the extranuclear ERα PIDs score obtained using the nearest-neighbor method and the true value (Pearson *r*^2^ = 0.91). (**D**) Typical imaging of the measurement of distance from the nucleus to the cell membrane. (**E**) Cell counts of 2,4,6, and 8 μm applied as the distance from nuclei to cell membrane by histogram analysis. Bar, 20 μm.

To verify the accuracy of the nearest-neighbor method, the cell membranes of patient samples representing a range of ERα expression levels were stained with an anti-sodium-potassium ATPase antibody-plasma membrane marker (PM-Marker) (Alexa Fluor^®^ 647) (Abcam, Tokyo, Japan). An expert pathologist carefully delineated every nucleus along with the boundary (Figure 3A, left) and marked whole cell membranes (Figure 3A, right). Then, the extranuclear ERα PIDs score (the number of total ERα PID particles per cell minus the nuclear ERα PID particles per cell) was calculated with high accuracy and recorded as the true value (Figure 3B, right). Distances of 2, 4, 6, and 8 μm were applied as the distance from the nuclei to the cell membrane using the nearest-neighbor method (Figure 3B, left). Then, the correlation between the extranuclear ERα PIDs score obtained using the novel method and the true value was evaluated. The extranuclear ERα PIDs score obtained using a distance of 4 μm in the nearest-neighbor method exhibited the strongest correlation to the true value (Pearson *r*^2^ = 0.91) (Figure 3C). The distance from the nuclei to the cell membrane was measured (Figure 3D), and the greatest number of cells exhibited a distance of 4 μm when the numbers of cells with distances of 2, 4, 6, and 8 μm were evaluated by histogram analysis (Figure 3E). Hence, 4 μm was chosen as the optimal algorithm for the nearest-neighbor method. This result indicated that the nearest-neighbor method provides an accurate measurement of the extranuclear ERα protein expression level.

### 2.4. Correlation between Biological Factors and Clinical Parameters of Patients

The patient characteristics and clinicopathological features are shown in Table 1 [25]. The median age at diagnosis was 52 years (range, 32–69 years). The corresponding lymph node metastases of the remaining 27 (41.5%) patients were included in this study. All 65 patients received adjuvant endocrine therapy after surgery, and 19 patients (29%) received adjuvant chemotherapy. The median follow-up time was 11.2 years (range, 1.3 to 15.8 years), and 21 (32%) patients developed relapse during the follow-up period. The scores for the proportion of stained cells and the staining intensity were summed to obtain the Allred score at diagnosis [26]. Patient tissue sections were re-stained by DAB-based conventional ERα IHC using standard procedures at Tohoku University Hospital (Sendai, Japan) with an anti-ERα rabbit mAb (SP1, Ventana, Tokyo, Japan) and an anti- progesterone receptor (PR) rabbit mAb (1E2, Ventana, Tokyo, Japan). The ERα/PR receptor status was reported using a semiquantitative H-score (from 0 to 300) determined with an Aperio image analysis system (Leica, Tokyo, Japan); the resulting score was strongly correlated with the manual score obtained by an expert pathologist (Supplementary Materials, Figure S2) [27]. The evaluation was performed, and the cut-off value of Ki67 LI (%) was determined as described in a previous report [28].

**Figure 4:**
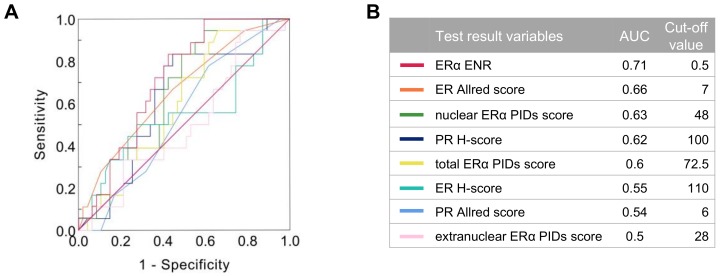
(**A**) Receiver-operating characteristic (ROC) curve analysis of biological factor scores between 65 patients with recurrence in 10 years and those without recurrence. (**B**) Area under the curve (AUC) and optimal cut-off values for each biological factor.

To investigate correlation between biological factors and the clinical parameters of patients, we evaluated the ER Allred score, ER H-score, PR Allred score, PR H-score, total ERα PIDs score, extranuclear ERα PIDs score, nuclear ERα PIDs score, and ERα ENR by setting cut-off values. The optimal cut-off values for each biological factor were determined by receiver-operating characteristic (ROC) curves based on the highest sum of sensitivity and specificity for BC recurrence within ten years (Figure 4A). The ERα ENR displayed the strongest ability to discriminate recurrent patients from nonrecurrent patients, with an area under the curve (AUC) of 0.71 (Figure 4B). We chose 0.5 as the best cut-off value of the ERα ENR for predicting recurrence, yielding a sensitivity of 83.3% and a specificity of 57.5%. However, an ERα ENR <0.5 or ≥0.5 was not associated with menopausal status, lymph node status, tumor stage, histological grade, PR H-score, or Ki67 LI (%) but was significantly associated with ER H-score (*p* = 0.017) (Table S1). When analyzing biological factors in recurrent and non-recurrent patients, we observed that the PR H-score (χ^2^ = 5.44, *p* = 0.02), total ERα PIDs score (χ^2^ = 4.44, *p* = 0.035), and nuclear ERα PIDs score (χ^2^ = 6.57, *p* = 0.01) were significantly negatively correlated with relapse. In contrast, the ERα ENR was significantly associated with relapse (χ^2^ = 6.23, *p* = 0.013) (Table 2).

**Figure 5:**
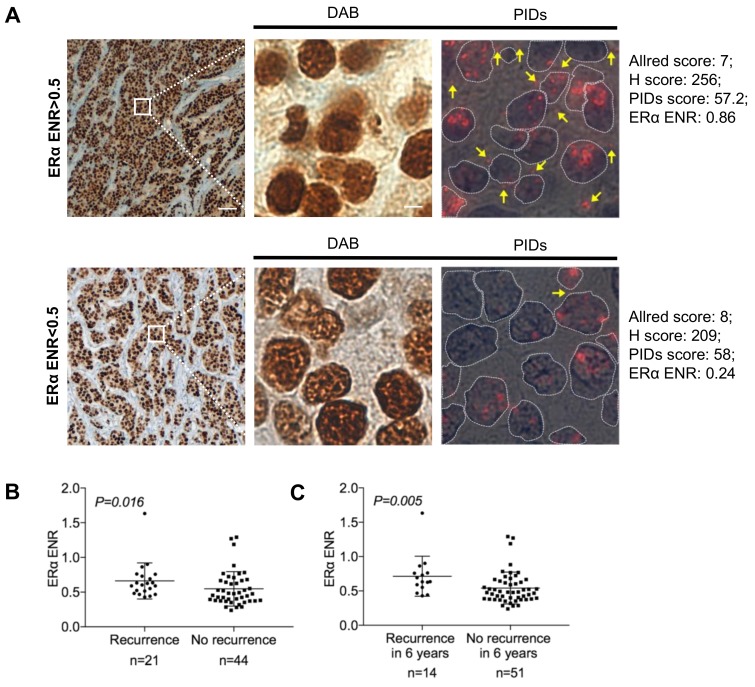
The correlation of the ERα ENR with recurrence and 6-DFS. (**A**) Conventional IHC-DAB (diaminobenzidine) and IHC-PIDs of two typical samples (top: recurrence; bottom: no recurrence). Two samples have similar Allred score, H score, and PIDs score of ERα expression, but they have opposite outcome. (**B**) Box plots with the continuous ERα ENR on the y-axis and recurrence or no recurrence on the x-axis. (**C**) Box plots with the continuous ERα ENR on the y-axis and 6-DFS on the x-axis.

Figure 5A shows two DAB images demonstrating similar Allred scores, H scores, and PIDs scores for ERα expression, but the sample from a recurrent patient had a higher ERα ENR than that from a nonrecurrent patient. These results show that the ERα ENR may better distinguish patients who may relapse compared to other conventional IHC-DAB measurements. Even ENR was not significantly different between the MCF-7 cells and EDR (estrogen-deprivation-resistant) cells in vitro (Figure S4). The ERα ENR was correlated with DFS and 6-DFS when measured as a continuous quantitative score (Figure 5B–C; DFS *p* = 0.016; 6-DFS *p* = 0.005).

### 2.5. Relationship between the Indexes of ERα Expression and Patient Survival

**Figure 6:**
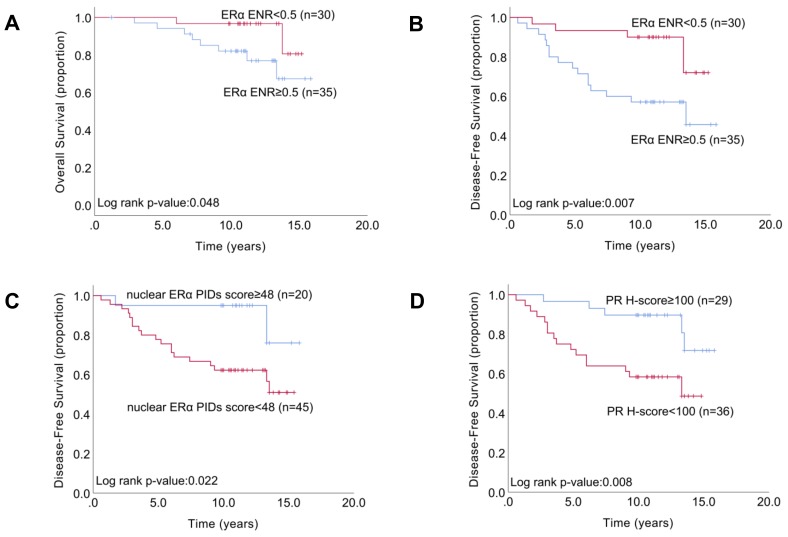
Kaplan–Meier analysis of overall survival (OS) according to the cut-off values of the ERα ENR (**A**). Kaplan-Meier analysis of disease-free survival (DFS) according to the cut-off values of the ERα ENR (**B**), nuclear ERα PID score (**C**), PR H-score (**D**). *p* values were determined with the log-rank test.

Based on the optimal cut-off values of the ER Allred score (6), PR H-score (100), ER H-score (110), total ERα PIDs score (72.5), extranuclear ERα PIDs score (28), nuclear ERα PIDs score (48), and ERα ENR (0.5), 65 patients with HR+/HER2- breast tumors who received ET were classified into two subgroups. Kaplan–Meier curves of estimated overall survival (OS) and DFS were generated, and comparisons between the two groups were performed with the log-rank test. Statistical analysis demonstrated that an ERα ENR ≥ 0.5 was strongly associated with decreased OS (log-rank *p* = 0.048; Figure 6A) and decreased DFS (log-rank *p* = 0.007; Figure 6B) compared to an ERα ENR < 0.5. Moreover, the 6-DFS rate was 65.7% for patients with a high ERα ENR (*n* = 35) and 93.3% for those with a low ERα ENR (*n* = 30) (log-rank *p* = 0.007; Figure 6F). In addition, a nuclear ERα PIDs score ≥ 48 and a PR H-score ≥ 100 were significantly associated with increased DFS compared to a nuclear ERα PIDs score <48 (log-rank *p* = 0.022; Figure 6C) and a PR H-score < 100 (log-rank *p* = 0.008; Figure 6D), respectively. In contrast, DFS was not correlated with the ER Allred score, ER H-score, total ERα PIDs score, extranuclear ERα PIDs score, or Ki67 LI (Supplementary Materials, Figure S3). Univariate analysis demonstrated a significant association between improved DFS and a high (≥48) nuclear ERα PIDs score (*p* = 0.01), high (≥72.5) total ERα PIDs score (*p* = 0.031), high (≥100) PR H-score (*p* = 0.008), low (<0.5) ERα ENR (*p* = 0.007), and lymph node status (*p* = 0.005) (Table 3). Multivariate analysis revealed that the ERα ENR was an independent prognostic factor of DFS (hazard ratio, 3.8; 95% CI, 1.4–11.8; *p* = 0.006), as were the PR H-score (hazard ratio, 3.9; 95% CI, 1.5–12.5; *p* = 0.006), and lymph node status (hazard ratio, 3.2; 95% CI, 1.3–8.7; *p* = 0.009) (Table 3).

## 3. Discussion

In the present study, IHC-PIDs analysis with the nearest-neighbor method was used to detect and quantitatively analyze ERα expression in both nuclear and extranuclear regions, resulting in higher sensitivity and specificity than conventional IHC-DAB. Additionally, ERα expression as assessed by IHC-PIDs in cell lines was strongly associated with the ERα protein levels quantified by flow cytometry and the ERα mRNA expression levels determined by real-time qRT-PCR. By estimating extranuclear ERα expression using the nearest-neighbor method, we obtained the ERα ENR and showed that a high ERα ENR was associated with poorer survival (OS and DFS) and remained an independent unfavorable factor for DFS in multivariate analyzes of patients in this study.

In current clinical practice, ERα is usually identified as a nuclear receptor; the clinical significance of extranuclear ERα has previously been neglected [29]. Despite accumulating evidence in preclinical studies suggesting that extranuclear ERα underlies endocrine resistance, no detailed evidence has been obtained from actual BC cases [30,31]. In this study, PIDs staining using SP1 indicated that PIDs could recognize extranuclear ERα in cultured cells and cell blocks. Thus, we developed a new computerized method using a nearest-neighbor method to separately evaluate nuclear and extranuclear ER expression. This study is the first to detect extranuclear ERα in tissue sections at such a high incidence in nearly all tumors examined (PIDs score range, 3 to 48).

Due to the possible opposing functions of nuclear and extranuclear ERα regarding ET resistance, we applied a novel scoring model defined as the ERα ENR [31,32]. This study is also the first to examine the relationship between the ratio of extranuclear ERα to nuclear ERα and patient outcomes. We report here that patients with *HR*^+^/HER2^−^ BC with relatively high expression levels of extranuclear ERα are less likely to benefit from ET than patients without these characteristics. ET resistance was defined as patients who relapsed after the first two years of ET (primary resistance) or as patients who relapsed within one year after the completion of five-year adjuvant treatment (acquired resistance) [5]. The continuous quantitative score for the comparison of the ERα ENR between patients with and without disease relapse at six years, defined as ET resistance, indicated that a high ERα ENR was a predictive factor for ET resistance. In addition, a high ERα ENR was associated with poorer survival and remained an independent unfavorable factor for DFS in the multivariate analyses of these patients. Extranuclear estrogen signaling has been linked to general activation of signaling pathways such as the MAPK/ERK, PI3K/AKT/mTOR, and protein kinase C pathways [33]. According to our research, we propose a hypothesis that even after treatment of tamoxifen or aromatase inhibitors, an increasing ERα ENR enable via non-genomic pathways dominates and induces the proliferation and survival of BC cells. Recently, clinical studies have evaluated cyclin-dependent kinase (CDK4/6) or mTOR inhibitors combined with hormonal treatment for patients who exhibit endocrine resistance [34,35]. Our study suggests that the early identification of patients with a high ERα ENR of breast tumor cells, who may be resistant to ET, could lead to the inclusion of a CDK4/6 inhibitor or mTOR inhibitor in the treatment regimen [36].

Although gene expression quantification technologies such as Oncotype DX have been shown to have predictive value in terms of BC outcomes [37,38,39], our study demonstrated that IHC-PIDs could be used as a complementary diagnostic tool to predict the response to ET in HR-positive HER2-negative breast cancer. In this study, our IHC-PID method showed higher sensitivity and specificity in a quantitative analysis than conventional IHC-DAB, and morphological information was retained, in contrast to other proteome analysis methods such as flow cytometry and western blotting. Furthermore, the PID score is strongly associated with the transcriptomic signature, implying that it could be applied before gene expression quantitation technologies. Notably, accumulating studies have suggested that not all protein levels exhibit a significant correlation with mRNA abundance [40,41,42]. Thus, the extremely high correlation between protein expression and mRNA expression is partially due to the use of a cell line rather than a patient specimen as the sample. 

By estimating ERα expression, we showed that an increased ERα PID score was associated with prolonged DFS. This finding is concordant with those of recent clinical studies showing that ERα status determined by IHC is predictive of patient responses to ET [3]. Furthermore, the association between an increased PR H-score and increased DFS is consistent with the results of clinical studies showing that the PR status determined by IHC is predictive of BC outcome [43]. In contrast, similar data were not obtained for the ER H-score or Allred score, which might be due to the small sample size.

An increasing number of research studies have suggested that in addition to the classical full-length 66-kDa ERα (ERα66) protein, which harbours two activation domains, namely AF-1 and AF-2, two other isoforms of 46 kDa (ERα46) and 36 kDa (ERα36) exist. ERα36 differs from ERα66 in that it lacks both the transcriptional activation domains (AF-1 and AF-2) and encodes a unique 29-amino-acid sequence. ERα36 is suggested to predominantly localize to the plasma membrane and cytoplasm and to mediate membrane-initiated estrogen signaling [44,45]. ERα46 lacks the N-terminal region containing the transactivation domain AF-1 [46]. As SP1 is reported to recognize the C-terminal domain of ERα, the extranuclear ERα that we detected in this study might be ERα46 or ERα66, implying that ERα46 and ERα66 might play essential roles in rapid signaling in BC.

There are several limitations of this study that should be considered. First, the boundary of each nuclei was manually determined by a pathologist, and although this procedure was performed carefully, artefacts cannot be avoided. Second, the high incidence of extranuclear ERα positivity might partially result from nonspecific immunostaining by PIDs due to their extremely high sensitivity, although we made efforts to reduce nonspecific binding in the experiments. Third, regarding the 65 HR+/HER2- BC specimens, the sample size and the retrospective analysis of this study have methodological limitations. 

Nevertheless, the use of IHC-PIDs provides new insight into this rare biomarker, and this study is the first to demonstrate the predictive value of extranuclear ERα for ET resistance in HR+/HER2- BC patients.

## 4. Materials and Methods

### 4.1. Patient Samples

This study was approved by the Ethics Committee of the Graduate School of Medicine at Tohoku University (No. 2015-1-311). We reviewed the data of 244 BC patients who underwent surgery at Tohoku University Hospital (Sendai, Japan) between 1 January 2001 and 31 December 2003 and enrolled 65 eligible patients according to the inclusion and exclusion criteria of this study. The inclusion criteria included the following: (1) aged between 18 and 70 years, without any other malignant tumors before initial diagnosis of BC; (2) pathologically confirmed ER-positive or/and PR-positive and HER2-negative BC patients; (3) received at least five years of ET; (4) essential clinicopathological status and follow-up information were available, including tumor size, tumor location, lymph node status, and histological grade; (5) patients had not received neoadjuvant chemotherapy. 

### 4.2. Cell Lines and Paraffin Sections

Five BC cell lines and one cervical cancer cell line covering a range of ER expression statuses were obtained from the American Tissue Culture Collection (ATCC). MDA-MB-231 and MCF-7 cells were cultured in phenol red-free Dulbecco’s Modified Eagle’s Medium (DMEM, Gibco, Life Technologies, CA, USA). ZR-75-1, BT-474, T47D, and HeLa cells were cultured in phenol red-free RPMI1640 medium (Gibco, Life Technologies, CA, USA). All media were supplemented with 10% foetal bovine serum (FBS, Gibco, Life Technologies, CA, US), and the cells were cultured at 37 °C in a humidified atmosphere of 5% CO_2_ in air [47]. HeLa cells in 35-mm glass-bottom dishes were transfected with a GFP-ERα expression plasmid or GFP-ERαΔNLS (nuclear localization sequence) expression plasmid using Lipofectamine^®^ LTX with PLUS™ Reagent (Invitrogen, CA, USA) according to the manufacturer’s instructions. The GFP-ERα expression plasmid was constructed from a human ERα cDNA expression plasmid [48] and GFP expression plasmid, pCMX-SAH/Y145F, which was kindly supplied by Dr Umesono (Saitama Cancer Center). ERα cDNA was obtained from a human ERα cDNA expression plasmid and ligated to pCMX-SAH/Y145F downstream of GFP via a BamH I site. The GFP-ERαΔNLS expression plasmid was obtained by an inverse PCR method and lacked the ERα NLS region from Arg256 to Lys303. A total of 1.5 × 10^7^ cells from each sample were fixed in 4% paraformaldehyde for 10 min and embedded with 2% alginic acid, followed by paraffin block preparation. Three-micron sections were cut and mounted on glue-coated glass slides.

### 4.3. Immunofluorescence

Paraffin sections of transfected cells were heated for 15 min at 65 °C, deparaffinized in xylene and hydrated in graded alcohol and distilled water. Antigen retrieval was performed in Tris EDTA buffer (pH 9) for 40 min at 95 °C. Cells cultured in 35-mm glass-bottom dishes were fixed with 4% paraformaldehyde for 15 min at room temperature (RT) and permeabilized with 0.2% Triton X-100 for 15 min at RT. After the cells were washed in phosphate-buffered saline (PBS), endogenous peroxidases and nonspecific binding were sequentially blocked by incubation with an Endogenous Biotin Blocking Kit (Ventana, Tokyo, Japan) for 10 min and with 10% goat serum (Funakoshi, Tokyo, Japan) in PBS for 1 h at RT, respectively. Cells were immunostained with a primary anti-ERα rabbit mAb (SP1, Ventana, Tokyo, Japan) overnight at 4 °C. After washing, the samples were incubated for 30 min with a biotinylated goat anti-rabbit IgG secondary antibody (Southern Biotech, AL, USA) diluted 1:50 in Dako antibody diluent (Dako, Tokyo, Japan) or mixed with 5 μg/mL anti-GFP mAb-Alexa Fluor^®^ 488 (RQ2) (Rat) (MBL, Nagoya, Japan) (for paraffin sections). Samples were incubated with 0.02 nM PIDs for 2 h and then with DAPI for 10 min at RT. After the final wash, the samples were mounted in Prolong™ gold antifade reagent (Invitrogen, CA, USA), and fluorescence images were acquired using a confocal laser scanning microscope (LSM 780, Carl Zeiss, Germany) and N-SIM (Structured Illumination Microscopy, Nikon, Tokyo, Japan).

### 4.4. IHC-PIDs

Formalin-fixed, paraffin-embedded (FFPE) cell lines and patient tissue sections were stained with an anti-ERα antibody as described above, and nuclei were stained with haematoxylin and mounted in Malinol mounting medium (Muto Pure Chemicals, Tokyo, Japan). The fluorescence signals of PIDs were observed using fluorescence microscopy (BX53, Olympus) with a UPLSAPO 40 × 2 (Olympus) objective lens and charge-coupled device (CCD) camera (DP73, Olympus) in 5 microscopic fields (with 1000 cells investigated in each sample). Then, the PIDs score was determined (PIDs per cell) [21].

### 4.5. Real-Time qRT-PCR

Total RNA was extracted from cultured cells using the TRIzol™ Plus RNA purification kit (Invitrogen, CA, USA) according to the manufacturer’s instructions. RNA (1 µg) was reverse transcribed to cDNA using the QuantiTect^®^ Reverse Transcription kit (Qiagen, Hilden, Germany). Real-time qRT-PCR was performed using Brilliant III Ultra-Fast SYBR^®^ Green QPCR Master Mix (Agilent Technologies, CA, USA) on a Step One™ Real-Time PCR System (Applied Biosystems, CA, USA) under optimal cycling conditions. The following primer sequences were used for ERα: forward, 5′-CTCCCACATCAGGCACAT-3′ and reverse, 5′-CTCCAGCAGCAGGTCATA-3′. Target gene expression was normalized to the expression of RPL13A, which was determined using the following primers: forward, 5′-CCTGGAGGAGAAGAGGAAAG-3′, and reverse, 5′-TTGAGGACCTCTGTGT ATTT-3′ [49].

### 4.6. Flow Cytometry

Single-cell suspensions of BC cell lines were obtained from cell cultures after trypsinization, fixed with Fixation Medium A (Invitrogen, CA, USA) for 15 min at RT and permeabilized with 0.2% Triton X-100 (Wako, Osaka, Japan) for 30 min at RT. Cells were stained with 1 μg/mL primary antibody to ERα (anti-ERα mouse mAb (1D5), Abcam, Tokyo, Japan) at RT for 20 min [30]. Afterward, flow cytometry was performed on a BD Aria II Flow Cytometer system (BD Biosciences, CA, USA) using a quantitative kit (QIFIKIT, Dako, CA, USA), which was used for the quantitative determination of cell antigen in accordance with the manufacturer’s instructions. Two washes were performed at each step with ice-cold Hanks’ Balanced Salt Solution (HBSS, Gibco, Life Technologies, CA, USA) containing 2% FBS. SP1 was used for flow cytometry to quantify IHC-PIDs and determine the amount protein, but the quantitative kit used for flow cytometry could only recognize the mouse antibody, thus 1D5 was used in this study.

### 4.7. Automatic Measurement of Nuclear and Extranuclear ERα

A novel, automatic, computerized measurement, termed the nearest-neighbor method, was developed to evaluate the expression of nuclear and extranuclear ERα using a PID analyzer (Konica Minolta, Tokyo, Japan). The nuclear boundary was carefully delineated by an expert pathologist in bright field (BL) of double stained images (Figure 3A, left), then the extranuclear PID particles were automatically recognized by selecting an optimal algorithm (Figure 3B, left). This method was capable of assessing the PIDs score of extranuclear ERα (the average PID particles per whole cell of ERα minus the average PID particles per nucleus of ERα). The extranuclear-to-nuclear ratio of ERα (ERα ENR) was calculated as the PID score of extranuclear ERα divided by the PID score of nuclear ERα. 

### 4.8. Statistical Analysis

Statistical analysis was performed with GraphPad Prism 7.0 (GraphPad Software) and JMP Pro 13 software (SAS Institute, Inc., Cary, NC, USA). Unpaired t-tests and one-way ANOVA were used to analyze differences in ERα expression among cell lines. The Pearson correlation test was used to examine the relationships among ER expression levels. Significant differences in clinicopathological features between groups were evaluated using either the χ^2^ test or t-test. Disease-free survival (DFS) was defined as the time from the date of surgery to first recurrence or death from BC without a recorded relapse. Six-year DFS (6-DFS) was assessed to evaluate the correlation between biological factors and endocrine resistance (recurrence while on ET but after the first 2 years of ET or within a year after completion of 5-year adjuvant treatment) [5]. Mann-Whitney U tests were used to determine the correlations between the continuous ERα ENR and DFS and 6-DFS. Survival estimates were obtained using Kaplan-Meier curves, and differences were evaluated with the log-rank test. To identify independent prognostic factors, backward stepwise multivariate Cox regression analyses were performed employing covariates that were significantly associated with DFS in the univariate analysis. Hazard ratios and their 95% confidence intervals (CIs) were calculated for each factor. *p* values were 2-tailed, and *p* < 0.05 indicated statistical significance.

## 5. Conclusions

In conclusion, we have developed a highly sensitive and specific quantitation method for extranuclear ERα, which was previously infrequently detected. We demonstrated a new method for localizing and assessing extranuclear protein that can be applied to other biomarkers to explore their underlying mechanisms. Furthermore, our results demonstrate that HR^+^/HER2^−^ BC with a high ERα ENR is less likely to benefit from ET. The nuclear ERα PIDs score also showed higher prognostic value than the total ERα PIDs score or H-score detection by IHC-DAB. In the future, we look forward to further prospective studies to confirm the results found here, using larger sample sizes to explore the implementation and evaluate the performance of this novel measurement in clinical use.

## Figures and Tables

**Table 1:** Patient characteristics.

Characteristics	Patients N (%)	Characteristics	Patients N (%)
**Age**		**Ki67 LI (%)**	
<50	25(38)	<14	44 (68)
≥50	40(62)	≥14	21 (32)
**Stage** ^1^		**Histological grade**	
I	30 (46)	1	20 (31)
II	29 (45)	2	34 (52)
III	6 (9)	3	11 (17)
**Lymph node metastasis**		**Adjuvant chemotherapy**	
Positive	27 (42)	+	19 (29)
Negative	38 (58)	-	46 (71)

^1^ Stage distribution is based on TNM classification of malignant tumors seventh edition by the International Union Against Cancer (UICC) (30).

**Table 2:** Correlations between biological factors and recurrence data.

Characteristics	No. (%) of Patients		*p* Value ^1^
	No Recurrence	Relapse	
	(*n* = 44)	(*n* = 21)	
**ER Allred score**			
<6	21 (32)	12 (18)	
≥6	21 (35)	9 (14)	0.477
**PR Allred score**			
<6	27 (42)	16 (25)	
≥6	17 (26)	5 (8)	0.237
**Ki67 LI (%)**			
<14	32 (49)	13 (20)	
≥14	12 (18)	8 (12)	0.377
**ER H-score**			
<110	13 (20)	9 (14)	
≥110	31 (48)	12 (18)	0.289
**PR H-score**			
<100	20 (31)	16 (25)	
≥100	24 (37)	5 (8)	0.02
**total ERα PIDs score**			
<72.5	29 (45)	29 (29)	
≥72.5	15 (23)	2 (3)	0.035
**nuclear ERα PIDs score**			
<48	26 (40)	19 (29)	
≥48	18 (28)	2 (3)	0.01
**extranuclear ERα PIDs score**			
<28	33 (51)	20 (31)	
≥28	11 (17)	1 (2)	0.049
**ERα ENR**			
<0.5	25 (38)	5 (8)	
≥0.5	19 (29)	16 (25)	0.013

^1^: Pearson χ^2^ test.

**Table 3:** Univariate and multivariate analysis of variables in the prediction for recurrence.

Characteristics	Univariate			Multivariate		
	Hazard Ratio	95% CI	*p* Value	Hazard Ratio	95% CI	*p* Value
**Lymph node metastasis**						
N ≥ 1/N0	3.5	1.5–9.2	0.005	3.2	1.3–8.7	0.009
**ER status (A score)**						
<6/≥6	1.4	0.6–3.5	0.407			
**PR status (A score)**						
<6/≥6	2.2	0.8–6.7	0.115			
**Ki67 IL (%)**						
≥14/<14	1.5	0.6–3.5	0.4			
**ER H-score**						
<110/≥110	1.6	0.6–3.8	0.307			
**PR H-score**						
<100/≥100	3.5	1.4–10.8	0.008	3.9	1.5–12.5	0.006
**total ERα PIDs score**						
<72.5/≥72.5	3.8	1.1–24.1	0.031			
**nuclear ERα PIDs score**						
<48/≥48	4.8	1.4–30	0.01			
**extra-nuclear ERα PIDs score**						
<28/≥28	4.9	1–87.3	0.048			
**ERα ENR**						
≥0.5/<0.5	3.6	1.4–11	0.007	3.8	1.4–11.8	0.006

## Supplementary Materials

The following are available online at https://www.mdpi.com/2072-6694/11/4/526/s1, Figure S1: Verification of transfection with GFP-ERα or GFP-ERαΔNLS expression plasmids, Figure S2: H-scores for the assessment of ER and PR are highly reproducible, Figure S3: Kaplan–Meier analysis of DFS according to ERα expression, Figure S4: A comparation of ENR between EDR cell and MCF-7 cell, Table S1: Associations between ERα ENR expression and clinicopathological characteristics.

## Abbreviations

ERα: estrogen receptor α; BC: breast cancer; ET: endocrine therapy; PIDs: phosphor-integrated dots; IHC: immunohistochemistry; SEM: scanning electron microscopy; CCD camera: Charge-Coupled Device camera; ENR: extranuclear-to-nuclear ratio; OS: Overall survival; DFS: disease-free survival; HR: hormone receptor; EREs: estrogen response elements; GFs: growth factors; RTKs: receptor tyrosine kinases; IGFR: insulin-like growth factor receptor; HER2: human epidermal growth factor receptor 2; DAB: diaminobenzidine; Qdots: quantum dots; FFPE: formalin-fixed, paraffin-embedded; PR: progesterone receptor; GFP: green fluorescent protein; NLS: nuclear localization sequence; RT: room temperature; PBS: phosphate-buffered saline; DAPI: 4’,6-diamidino-2-phenylindole dihydrochloride; real-time qRT-PCR: real-time quantitative reverse transcription-PCR; CLSM: confocal laser scanning microscope; N-SIM: Structured Illumination Microscopy; 6-DFS: six-year DFS; CIs: confidence intervals; ROC: receiver-operating characteristic; AUC: area under the curve; LI: Labeling index; EDR: estrogen-deprivation-resistant.
