# Supplementary material for: Automated Quantification of Extranuclear ERα Using Phosphor-Integrated Dots for Predicting Endocrine Therapy Resistance in HR+/HER2− Breast Cancer
Source: Cancers (Basel). 2019 Apr 12;11(4):526. doi: 10.3390/cancers11040526 (PMC6520781; doi:10.3390/cancers11040526)
Supplement: Supplementary file 1 [file cancers-11-00526-s001.pdf]

cancers

Supplementary Material: Automated quantification of extranuclear ERα using phosphor-integrated dots for predicting endocrine therapy resistance in HR+/HER2- breast cancer

Zhaorong Guo, Hiroshi Tada, Narufumi Kitamura, Yoh Hamada, Minoru Miyashita, Narumi Harada, Akiko Sato, Yohei Hamanaka, Kouki Tsuboi, Nobuhisa Harada, Mayumi Takano-Kasuya, Hisatake Okada, Yasushi Nakano, Noriaki Ohuchi, Shin-ichi Hayashi, Takanori Ishida[1] Kohsuke Gonda

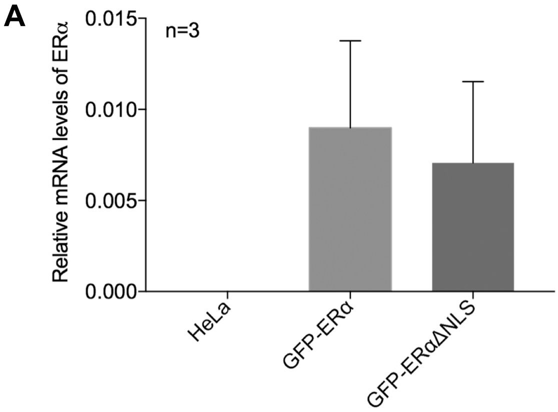

Figure S1. Verification of transfection with GFP-ERα or GFP-ERαΔNLS expression plasmids**.** Relative copy number of ERα mRNA expression level for HeLa cells, GFP-ERα plasmids-transfected HeLa cells, GFP-ERαΔNLS plasmids-transfected HeLa cells.

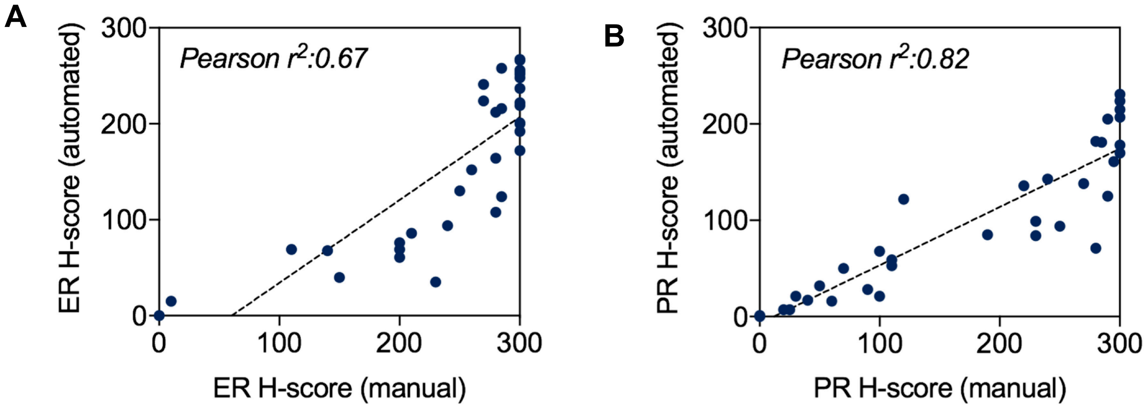

Figure S2. H-scores for the assessment of ER and PR are highly reproducible. The H-scores of randomly selected 34 cases were assessed with the Aperio image analysis system, which has a strong correlation with manual scoring evaluation by an expert pathologist. Pearson coefficients r^2=0.67 (ER, **A**) and 0.82 (PR, **B**).

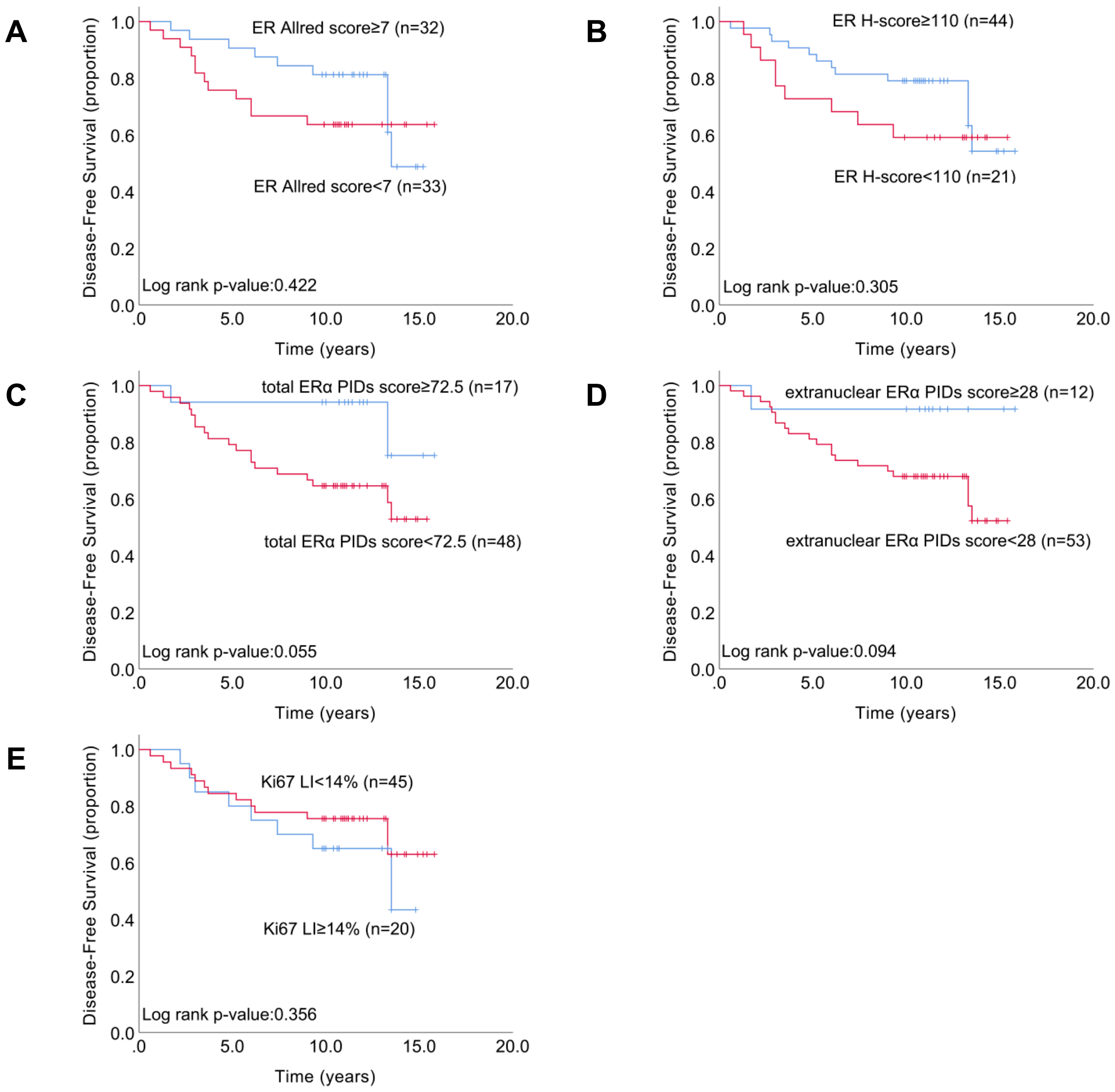

Figure S3. Kaplan-Meier analysis of DFS according to ER*α* expression. Kaplan-Meier analysis of DFS according to the cut-off values of the ER Allred score(**A**), ER H-score (**B**), total ER*α* PID score (**C**), extranuclear ER*α* PID score (**D**), Ki67 LI (**E**). *P* values were determined with the log-rank test.

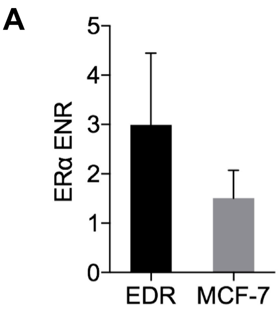

Figure S4 A comparation of ENR between EDR (estrogen-deprivation-resistant) cell and MCF-7 cell (*p= 0.066*)

.

Supplementary Table S1. Associations between ERα ENR expression and clinicopathological

**Characteristics**	**No. (%) of Patients**		***P value***
	**ERα ENR<0.5 (n=30)**	**ERα ENR≥0.5 (n=35)**	
**Menopausal status**			
pre	7 (11)	21 (32)	
post	11 (17)	26 (40)	*0.673*
**Lymph node status**			
N0	12 (18)	26 (40)	
N≥1	6 (9)	21 (32)	*0.406*
**Stage**			
I	9 (14)	20 (31)	
II	8 (12)	21 (32)	
III	1 (2)	6 (9)	*0.674*
**Histological grade**			
1	6 (9)	14 (22)	
2	9 (14)	25 (38)	
3	3 (5)	8 (12)	*0.961*
**Ki67 LI (%)**			
<14	13 (20)	32 (49)	
≥14	5 (8)	15 (23)	*0.746*
**ER H-score**			
<110	2 (3)	20 (31)	
≥110	16 (25)	27 (41)	*0.017*
**PR H-score**			
<100	11 (17)	25 (38)	
≥100	7 (11)	22 (34)	*0.565*

© 2019 by the authors. Submitted for possible open access publication under the terms and conditions of the Creative Commons Attribution (CC BY) license (http://creativecommons.org/licenses/by/4.0/).
